# Affective Video Retrieval: Violence Detection in Hollywood Movies by Large-Scale Segmental Feature Extraction

**DOI:** 10.1371/journal.pone.0078506

**Published:** 2013-12-31

**Authors:** Florian Eyben, Felix Weninger, Nicolas Lehment, Björn Schuller, Gerhard Rigoll

**Affiliations:** 1 Institute for Human-Machine Communication, Technische Universität München, München, Germany; 2 Department of Computing, Imperial College, London, United Kingdom; ICREA-University of Barcelona, Spain

## Abstract

Without doubt general video and sound, as found in large multimedia archives, carry emotional information. Thus, audio and video retrieval by certain emotional categories or dimensions could play a central role for tomorrow's intelligent systems, enabling search for movies with a particular mood, computer aided scene and sound design in order to elicit certain emotions in the audience, etc. Yet, the lion's share of research in affective computing is exclusively focusing on signals conveyed by humans, such as affective speech. Uniting the fields of multimedia retrieval and affective computing is believed to lend to a multiplicity of interesting retrieval applications, and at the same time to benefit affective computing research, by moving its methodology “out of the lab” to real-world, diverse data. In this contribution, we address the problem of finding “disturbing” scenes in movies, a scenario that is highly relevant for computer-aided parental guidance. We apply large-scale segmental feature extraction combined with audio-visual classification to the particular task of detecting violence. Our system performs fully data-driven analysis including automatic segmentation. We evaluate the system in terms of mean average precision (MAP) on the official data set of the MediaEval 2012 evaluation campaign's Affect Task, which consists of 18 original Hollywood movies, achieving up to .398 MAP on unseen test data in full realism. An in-depth analysis of the worth of individual features with respect to the target class and the system errors is carried out and reveals the importance of peak-related audio feature extraction and low-level histogram-based video analysis.

## Introduction

Affective computing refers to emotional intelligence of technical systems in general, yet so far, research in this domain has mostly been focusing on aspects of human-machine interaction, such as affect sensitive dialogue systems [1]. In this light, audio and video analysis have been centered on the emotion conveyed by humans by means of speech, facial expressions and other signals such as non-linguistic vocalizations, posture etc. [2]. However, less attention has been paid to the affective information contained in general audio-visual recordings, although it is common sense that such information is ever-present—for example, if one thinks of a video of a pleasant landscape with singing birds, or a dark scene with the creeky sound of a door opening. Automatic prediction of affective dimensions of sound, for example, has been addressed in [3], [4] for general acoustic events, and more specifically in a large body of literature on ‘music mood’, as summarized by [5].

In general, endowing systems with the intelligence to describe general multi-modal signals in affective dimensions is believed to lend to many applications including computer aided sound and video design, summarization and search in large multimedia archives; for example, to let a movie director choose particularly ‘creepy’ sounds from a large library, or to let users browse for music or movies with a certain mood. Another use case is to aid parental guidance by retrieving the most ‘disturbing’ scenes from a movie, such as those associated with highly negative valence. As a special case, yet one of high practical relevance, automatic classification of violent and non-violent movie scenes has been studied.

This problem is commonly approached using multi-modal classification strategies based on visual and audio information. A good introduction to affective video content modeling is found in [6].

A fairly early study on violent scene characterisation is found in [7]. Three groups of visual descriptors are used: the spatio-temporal dynamic activity as an indicator for the amount and speed of motion, an audio-visual flame detector based on colour values, and a blood detector based on colour values. The acoustic classification consists of Gaussian modelling of the soundtrack, i.e., the overall auditory scene, as well as the energy entropy as a measure for sudden loud bursts [8]. in contrast focusses on human to human violence only and uses human limb trajectory information to estimate the presence of violence. Giannakopoulos et al. [9] present an approach for identifying violent videos on video sharing sites. They use a feature level fusion approach where they fuse 7 audio features with 1 visual feature: the percentage of shots shorter than 0.2 seconds. The 7 audio features are mid-term features: they are probabilities of a Bayesian network classifier for 7 audio classes such as music, speech, gunshots, etc. A 2 second window with 50% overlap is used thereby. Additionally a text-based feature is used which reflects the relatedness of the words in the comments for the video to words of violence.

Gong et al. [10] use a two-stage detection approach wherein a pre-selection of candidate shots is generated by low-level classifiers. These candidate shots are then examined further for pre-defined high-level audio events in order to arrive at a violence score. Lin et al. [11] use a combination of low-level audio and video features together with specialized detectors for high-level events such as flames, explosions and blood. The collected information is then combined in a co-training framework. This approach of combined low-level features with additional high-level detectors is also used by Giannakopoulos et al. [12]. In their work, low-level audio and video features are paired with a continous person tracking algorithm which generates an actor-specific motion score for each shot. Audio features are first used by an ‘One-vs-All’ classification to assign the shot a basic audio class which is then fused with the results of the video motion analysis in a k-nearest neighbor (kNN) binary classifier. The same basic idea of considering motion patterns within a shot is adapted by de Souza et al. [13] for a visual Bag-of-Words classification. In contrast to Giannakopoulos' approach, the spatio-temporal features are not limited to tracks of faces and persons but track any stable visual interest point. However, audio is not taken into account for classification purposes, thus deviating from the previous multi-modal approaches. The Bag-of-Words framework is also employed by Nievas et al. [14] who use a very similar technique based on MoSIFT features to classify ice hockey clips in a purely visual analysis. Chen et al. [15] build upon this previous work and try to constrain the definition of violence as a series of action followed by the appearance of blood. Accordingly, a combination of visual motion analysis and color-based, localized blood detection is used to drive a Support Vector Machine (SVM) based classifier. Wang et al. [16] have introduced a novel approach for visual violence detection on a data-set built by themselves. Their method is based on Discriminative Slow Feature Analysis (D-SFA) where slow feature functions are learnt from dense trajectories inferred from the motion in the videos. Support Vector Machines are used in the end to classify videos as violent or non-violent. All this work demonstrates particularly well a current major problem of violence detection in movies: Without an independent baseline dataset and a common definition of violence, comparisons between different approaches become practically meaningless.

Thus, to provide objective metrics of feature relevance and system performance in full realism, we evaluate our own system on the official corpus of the MediaEval 2012 campaign (Affect Sub-Task) consisting of 18 Hollywood movies extending over 35 hours of audio-visual material in total. This data set employs a broad definition of violence as ‘physical action that results in human injury or pain’. Consequently, we approach the violent scenes detection problem in a generic way. Instead of relying on hand-crafted detection of events related to a particular definition of violence, we leverage computational intelligence: We apply a machine learning centered processing chain, including pre-processing and automatic segmentation, large-scale ‘brute-force’ audio-visual feature extraction, classifier training and optimization, and score fusion. Our methodology is motivated from our previous research on affect recognition both from audio-visual recordings of human-computer interaction, and from general sound.

Our preliminary results with this approach have been promising [17]; yet, many of the practical issues that have been discussed in ‘traditional’ affective computing for human emotion, such as finding relevant features, appropriate segmentations, and meaningful evaluation measures, have to be addressed in more detail in the light of the new paradigm of general affective multimedia analysis—these considerations will be the focus of this article. In particular, an in-depth analysis of the worth of individual features, especially their relatedness to different types of system errors, will be carried out—such broad analysis has, to the best of our knowledge, never been attempted before for violence detection.

Starting from this broad picture, the remainder of this article will now provide a more precise description of the evaluation data set, the system components, and its performance. In the end, we provide performance bounds of our segmental feature extraction approach assuming manual pre-segmentation.

## Evaluation Database

Our approach is evaluated on the official data sets of the MediaEval 2012 Affect Task evaluation campaign [18], derived from 18 well-known Hollywood movies. The data is available upon request from Technicolor (https://research.technicolor.com/rennes/vsd/), and more details on the data set are given in [19]. The evaluation campaign was initiated by Technicolor France and has been based on the use case of parental guidance, where parents could have a system retrieve the most violent scenes in a movie, review them and then decide if the movie is suitable for their children, instead of blindly relying on the age rating or having to watch the entire movie in advance. The task of violent scenes detection is thereby evaluated on ‘shot level’: that is, a score has to be provided for every shot in order to create a ranked list of potentially violent ones. The shot boundaries have been automatically annotated by the challenge organizers based on a keyframe detection algorithm. Note that the violent scenes annotation is not aligned to any shot boundaries.

The annotation of the data set was performed at Technicolor France. To establish a ‘ground truth’ annotation, violence was defined as ‘physical violence or accident resulting in human injury or pain’. Seven human assessors were employed to create the annotation [18].

The list of movies is shown in Table 1. As can be seen, the data set covers movies from vastly different genres and mainly the past two decades, with the exception of The Wizard of Oz (1939, colour – artificially painted), and Midnight Express (1978). As a result, the data set provides a challenging ‘cross-database’ setup where classifiers and features have to generalize to various genres, recording quality, camera work from rather static perspectives in earlier movies to highly dynamic shooting in today's action movies, and the type of violence portrayed (e. g., gunfights, martial arts, or ‘magic’). The data is sub-divided into a development (15 movies) and test set (three movies). As one can see from Table 1, the average length of the automatically detected shots varies considerably; this is partly due to genre. Furthermore, the relative duration of scenes annotated as violent ranges from below one percent (*Dead Poets Society*) to over ten percent (*Kill Bill 1*). In fact, these two measures exhibit significant negative correlation (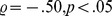 according to a two-sided t-test), indicating that ‘fast-paced’ movies also have more violent scenes, which is intuitive. This motivates the inclusion of a ‘segment duration’ feature for violent scenes detection whenever the segmentation by keyframes is used.

**Table 1:** MediaEval 2012 Affect Task Data Set: 18 Hollywood movies by title, year of recording, duration (Dur), number (#) of shots determined by automatic keyframe detection, average shot duration, and proportional length of violent scenes relative to movie duration in percent.

Title	Year	Dur [h∶m∶s]	# Shots	Avg. shot dur. [s]	Violence [%]	Fold
*Development Set*						
Armageddon	1998	2∶24∶40	3 562	2.4±6.5	5.8	1
Billy Elliot	2000	1∶45∶49	1 236	5.1±10.0	1.7	1
Eragon	2006	1∶39∶45	1 663	3.6±9.3	6.0	3
Harry Potter V	2007	2∶12∶33	1 891	4.2±9.6	3.9	3
I Am Legend	2007	1∶36∶19	1 547	3.7±9.6	7.6	3
Kill Bill 1	2003	1∶46∶10	1 597	3.9±12.4	10.1	1
Leon	1994	1∶45∶44	1 547	4.1±7.5	2.7	2
Midnight Express	1978	1∶56∶01	1 677	4.1±7.4	4.5	3
Pirates of the Caribbean	2003	2∶17∶19	2 534	3.2±7.5	5.7	2
Reservoir Dogs	1992	1∶35∶12	856	6.6±12.4	4.9	1
Saving Private Ryan	1998	2∶42∶30	2 494	3.9±7.4	8.5	2
The Bourne Identity	2002	1∶53∶36	1 995	3.4±9.3	3.3	2
The Sixth Sense	1999	1∶42∶58	963	6.4±13.4	1.0	1
The Wicker Man	2006	1∶37∶50	1 638	3.5±7.8	2.9	2
The Wizard of Oz	1939	1∶37∶39	908	6.4±10.3	1.8	3
*Test Set*						
Dead Poets Society	1989	2∶03∶33	1 583	4.6±7.2	0.7	-
Fight Club	1999	2∶13∶25	2 335	3.4±5.3	7.6	-
Independence Day	1996	2∶27∶14	2 652	3.3±7.3	6.4	-
Σ		35∶18∶24	32 678			

## Methodology

### 2.1 Audio-Visual Feature Extraction

Our feature extraction method is motivated from the domains of affect recognition from human speech and sound events [3], and general paralinguistic audio information retrieval [20], [21]. A large-scale feature set is ‘brute-foced’ by summarizing low-level descriptors (LLDs) extracted from short audio frames over segments of multiple frames. Within these frames, statistics such as mean, standard deviation, higher moments, quartiles, regression coefficients, etc. are applied to the LLDs. This way, LLD series of variable length can be mapped onto a single feature vector. The same approach is used for both audio and video features.

By that, it is evident that the choice of segments is a crucial issue. Naturally, we could summarise the LLD over each shot. The shots are provided by the automatic shot segmentation available in the MediaEval database. This segmentation method is referred to as *shot*. Since the shot lengths generally show very large standard deviations, alternative segmentations into fixed (maximum) length sub-windows of shots will be considered to provide more consistent functionals. In turn, when choosing the fixed segment length, one has to take into account that longer segments will contain more information, but possibly violence mixed with non-violence or simply different violent or non-violent content. Therefore, we divided each shot into sub-windows of a fixed maximum length. In pre-evaluation runs [17], we found that 2 seconds long sub-segments gave good results. In this study we now systematically investigate different segmentation methods and shot sub-windows. Here, we apply a range of sub-window lengths from 6, 4, 2, and 1 seconds. Furthermore, we investigated both overlapping sub-segments sampled at a rate of 1.0 seconds, and non-overlapping sub-segments. The motivation behind overlapping sub-segments is the increased amount and diversity of training data. Different alignments of the segments with the actual data are available for training the classifier, thus presumably making the results more stable.

Extraction of the acoustic features is done with our open-source feature extraction toolkit openSMILE [21]. We stick to rather simple spectral and energy based LLDs for the experiments in this article. We deliberately do not use any voice quality or pitch related descriptors, as these are obviously motivated by the presence of speech in paralinguistic audio analysis. While the movies do also contain speech, violent segments need not necessarily contain speech. These voice specific features are therefore not a reliable source of information for violence. While they might not be completely useless, we decided for straightforward features that can be extracted from any type of acoustic signal equally well. Clearly, energy or variants such as loudness could be indicative of scenes with high arousal. The RASTA-style filtered auditory spectrum sum (cf. Table 2) is a kind of loudness measure of events modulated with 4–8 Hz, i. e., a band-pass filter with a passband between 4 and 8 Hz is applied to the temporal envelopes of the auditory spectral bands. This bandwidth is motivated by the average modulation frequency of speech signals. We consider these features in addition since violence could co-occur with high-energy speech (screaming).

**Table 2:** Acoustic and visual low-level descriptors.

**4 acoustic energy LLDs**
Sum of auditory spectrum (loudness)
Sum of RASTA-style filtered auditory spectrum
Logarithmic energy, and zero-crossing rate
**33 acoustic spectral LLDs**
MFCC 1–16
Spectral energy 40–150, 250–650 Hz, 1 k–4 kHz, 5 k–15 kHz
Spectral roll-off point 0.25, 0.50, 0.75, 0.90
Spectral flux, entropy, variance, skewness, kurtosis,
slope, psychoacoustic sharpness, harmonicity, centroid
**95 visual LLDs**
Normalised HSV histograms (20, 20, 10 bins)
Normalised dense Optical Flow histograms (20 bins)
Normalised Laplacian edge histograms (20 bins)
Mean Optical Flow
Optical Flow standard deviation
Strongest edge in lower 98% of Laplacian edges

Furthermore, the distribution of energy to different frequency bands as well as related spectrum descriptors such as slope, centroid, variance, skewness, sharpness and harmonicity are expected to be of interest, to detect, e.g., broadband impact noises with high low-frequency content which could be indicative of gunshots or explosions. Spectral flux describes the amount of spectral change of two consecutive audio frames. Thereby both changes in frequency and signal energy are considered. Thus, both an amplitude modulated tone and a frequency modulated tone (with constant energy) would have a non-zero, positive spectral flux. The 37 acoustic LLDs, given in Table 2 are extracted from overlapping audio frames of 25 ms length, sampled at a rate of 10 ms.

First order delta coefficients are computed from the LLDs in order to better capture the dynamics of the input. 45 functionals (cf. Table 3) are applied to the acoustic LLDs and their first order delta coefficients. These functionals are standard in paralinguistic information retrieval and consist mainly of extrema, means and moments, percentiles, as well as temporal information; from the latter, we suspect especially peak- and slope-based analysis to be fruitful to capture important ‘highlights’ in the feature contours for violence detection. Gunshots, for example, would be characterized by rapidly rising and falling slopes in the energy contour. The total dimensionality of the acoustic feature set is 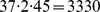.

**Table 3:** 45 functionals applied to acoustic and visual low-level descriptors and delta coefficients.

quartiles 1–3 and all 3 inter-quartile ranges
1% percentile (≈min), 99% percentile (≈max)
percentile range 1%–99%
position of min/max, range: max-min
arithmetic mean, root quadratic mean
contour centroid, flatness
standard deviation, skewness, kurtosis
rel. duration LLD is above 90%/below 25% of range
rel. duration LLD is rising/falling
range of peaks (absolute and rel. to arith. mean)
mean value of peaks (absolute and rel. to arith. mean)
mean value of peaks – arithmetic mean
mean value of minima rel. to arith. mean
max, min, mean, std. dev. of rising/falling slopes
mean/std.dev. of inter maxima distances
linear regression slope, offset, and quadratic error
quadratic regression coefficient 1, and quadratic error
duration of the underlying segment

The low level video features are computed for each frame and consist of Hue-Saturation-Value (HSV) histograms, an optical flow analysis and a Laplacian edge detection. Three, dimensionally independent, normalised HSV histograms (20, 20 and 10 bins) are computed. A dense Farneback optical flow analysis compares consecutive frames for pixel-wise displacements. The magnitudes of the resulting 2D displacement vectors are computed, thresholded to a maximum displacement of 15% of the normalised frame size and sorted into 20 bins. The resulting histogram is then normalised. Next, the mean optical flow and its standard deviation are determined. These frame-to-frame motions are expected to yield information concerning the overall pacing of the current scene. Furthermore, high standard deviations on optical flow would signify non-uniform scene flow while high mean flows could indicate a fast-paced scene. Finally, Laplacian edge detection is used for a simple detection of motion blur. An edge image is computed per frame, the 2% strongest edges are discarded as noise and the remaining strongest edge is used as a feature. Additionally, a normalised magnitude histogram of the edge image is calculated, ignoring values close to zero (histogram range: 16–255, 8-bit edge image). All 95 visual descriptors are given in table 2. First order delta coefficients are computed for all the visual LLD to capture temporal dynamics of the LLD. The same 45 functionals (cf. Table 3) as for the audio features are applied to the frame-wise visual LLDs and their first order delta coefficients with openSMILE in order to summarise the low-level descriptor features over windows of fixed (maximum) size. In this way, a total of 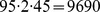 video features are obtained.

### 2.2 Feature Analysis

**Figure 1:**
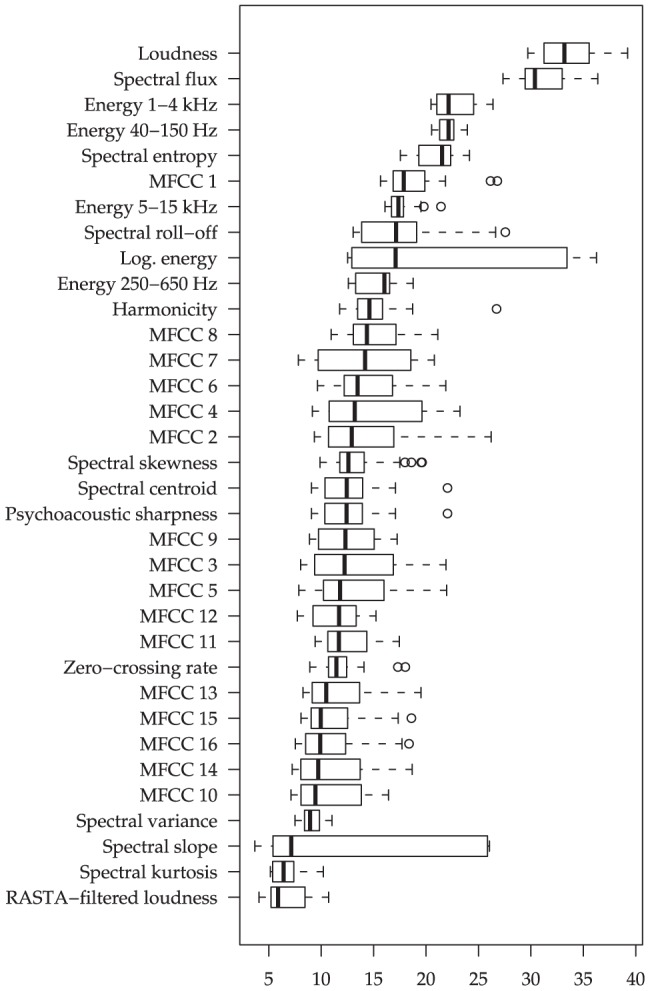
Audio LLD relevance: Absolute values of t-statistics (non-violent vs. violent) for groups of LLDs, across functionals applied to two second non-overlapping segments. 26/45 functionals selected by highest t-statistic per LLD group.

**Figure 2:**
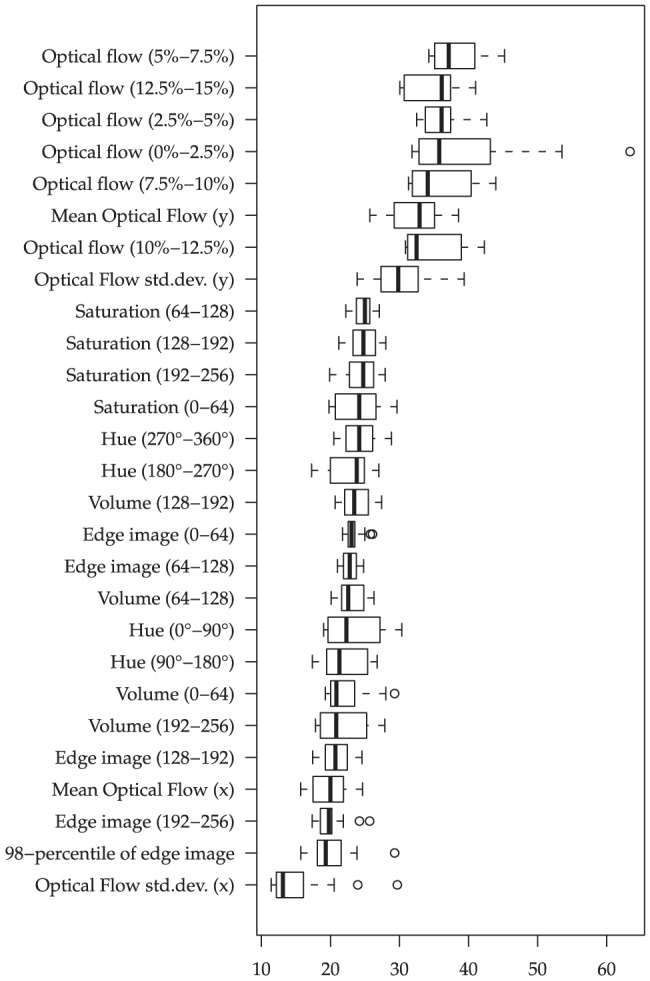
Video LLD relevance: Absolute values of t-statistics (non-violent vs. violent) for groups of LLDs, across functionals applied to two second non-overlapping segments. 26/45 functionals selected by highest t-statistic per LLD group.

To verify the soundness of the above feature extraction procedure independently of a classifier, we calculate the t-statistic with respect to the ‘violent’ and ‘non-violent’ windows for each individual feature. Windows are annotated as ‘violent’ whenever they coincide with a violent segment in the manual annotation. The t-statistics analysis was conducted on shot sub-segments of maximum length 2 seconds without overlap for both audio and video. To provide the ‘big picture’, in Figures 1 and 2 the absolute values of these t-statistics are visualized for different types of LLDs as box-and-whisker plots. Boxes range from the first to the third quartile and all values that exceed this range by more than 1.5 times the width of the box are considered as outliers; these are depicted by circles. For each LLD the t-statistics are average over the functionals. However, as we always find ‘inappropriate’ LLD/functional combinations that are of little relevance (t-statistic close to zero), only the ‘top half’ (wrt. t-statistics) of the functionals for each LLD are considered.

For audio features (Figure 1), the hypothesized importance of energy-related descriptors is confirmed. Among them, loudness seems to be particularly relevant while log-energy is somewhat inferior. Energy with the speech modulation frequency (sum of RASTA-style filtered spectrum) is not as indicative of violence; this is arguably due to the concept of ‘violence’ followed in the annotation, relating to physical violence only. Subdividing energy into frequency bands, we find middle to high frequencies (1–4 kHz) as well as low frequencies (40–150 Hz) to be most indicative while middle frequencies (250–650 Hz) are least discriminative; this observation can probably be attributed to the presence of loud broadband impact noises in violent scenes. The most important descriptors of the spectral distribution seem to be spectral flux, entropy, harmonicity, and skewness (in that order, by median absolute t-value). Related to these spectral distribution features, we now examine MFCC features, and find a mixed picture: Especially the first MFCC, which is somewhat similar to spectral skewness, is apparently relevant; however, some functionals of higher order MFCCs should be considered as well, such as peak distances of the 3rd to 6th MFCC—these distances are apparently much lower for non-violent scenes, indicating slower change of the general acoustic scene.

Interestingly, our findings on the visual channel corroborate these observations. Foremost, we observe features related to the optical flow ‘on top’ of the visual LLDs, corroborating the correlation between fast-paced scenes and violence hypothesized in the previous section. Among the single most important optical flow features are the minimum (1-percentile) and arithmetic mean of the 0%–2.5% histogram bin (t = 64 and t = 52, respectively, for non-violent vs. violent); furthermore, the rise times of the higher optical flow bins (i. e., corresponding to higher percentage of image dimension) are much lower in non-violent than in violent scenes, relating to stronger acceleration. Interestingly, the mean optical flow in y-direction seems to be much more relevant than the x-direction. Next to optical flow, the color-related features seem important to characterize violent scenes. However, it is important to note that mostly the change in color seems to be relevant, as among the most important descriptors we find, e. g., relative peak ranges of the 0–12 saturation bin (t = 29).

**Figure 3:**
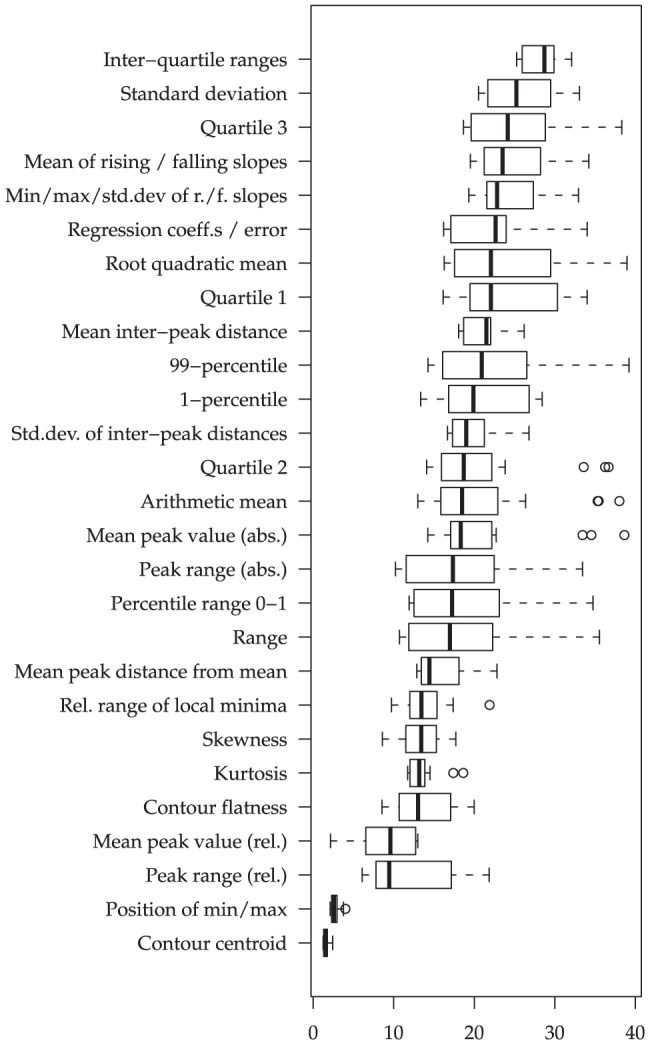
Functional Relevance for audio: Absolute values of t-statistics (non-violent vs. violent) for groups of functionals applied to two second non-overlapping segments, across audio/video LLDs. 18/37 (audio) and 48/97 (video) LLDs selected by highest t-statistic per functional group.

**Figure 4:**
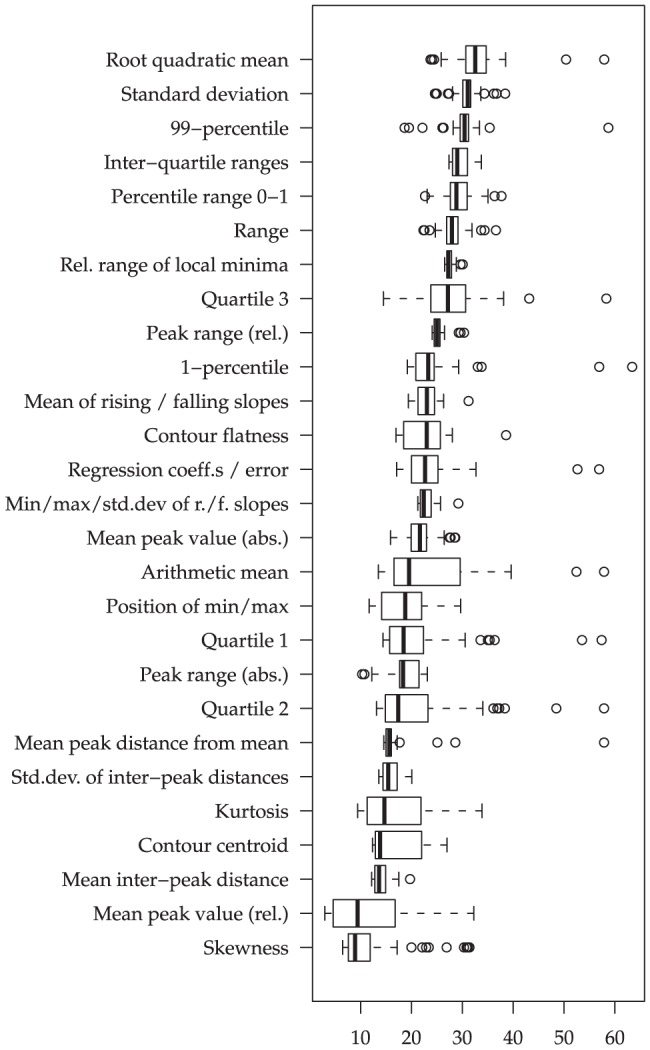
Functional Relevance for video: Absolute values of t-statistics (non-violent vs. violent) for groups of functionals applied to two second non-overlapping segments, across audio/video LLDs. 18/37 (audio) and 48/97 (video) LLDs selected by highest t-statistic per functional group.

Next, let us take a closer look at the importance of different functionals: In Figures 3 and 4, the t-statistics are summarized for the different functionals across the ‘better half’ of the LLDs. Among the functionals which seem most conducive to violence prediction from audio features (Figure 3) are the first and third quartile, which are more important than the overall median or mean. Furthermore, functionals related to peaks (local maxima), such as the statistics of the falling and rising slopes, seem highly relevant. Among the types of means applied, the root quadratic mean is particularly important, apparently because it considers rising and falling contours equally. For video features, we observe mostly ‘classical’ functionals such as means, moments and quartiles as relevant. Peak functionals do not seem as noteworthy as for the audio features; in fact, events related to peaks in the audio such as impact noises might not necessarily imply such peaks in the visual channel.

Overall, our results concerning audio LLDs are in accordance with previous findings in sound emotion recognition [3]; in particular, it is interesting that in the feature space, violence seems to be correlated with the arousal dimension often considered in sound and human emotion recognition (fast paced, ‘action-prone’ scenes). In this light, we also point out that the hypothesis put forth at the end of Section 1 is corroborated by the fact that the mean length of non-violent shots is significantly lower (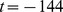, 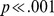).

### 2.3 Classification and Fusion

Our method for detection of violent scenes uses SVM classifiers which are trained on features extracted from the development data. Due to the large feature space, a linear kernel is chosen. Further, we did not evaluate other classifiers on this data for two reasons: 1) our past experience has shown that linear kernel SVM are on average by far the best classifier for such high dimensional feature vectors, both with respect to training time and accuracy; 2) we decided to keep the number of reported results low in order to not overwhelm the reader with lots of figures which are similar. Instead we wanted to focus on the feature analysis, as well as a discussion of the input segmentation. Independent classifiers are trained on acoustic and visual features.

During SVM training, logistic regression models are built on the hyperplane distances of the positive and negative training instances, in order to obtain a mapping to confidence scores in the interval from 0 to 1. The Sequential Minimal Optimization (SMO) algorithm implemented in the Weka toolkit [22] is used. Various complexity parameters C (influencing the number of randomly selected instances from the training data used to build the model) are investigated: 0.0005, 0.001, 0.005, and 0.01. To obtain a single decision and confidence score for each shot, the predictions made by the acoustic and visual SVMs are fused by simple score averaging (see the previous sub-section).

**Figure 5:**
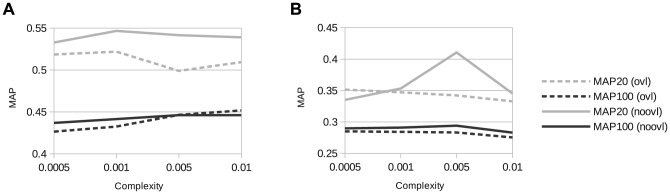
Segmentation and classfier training: Influence of SVM complexity and overlapping (ovl, 50%) vs. non-overlapping (noovl) shot sub-windows on (shot level) mean average precision (MAP), measured on the development set of the MediaEval 2012 Affect Task Data Set. Sub-window size 2 seconds. A: audio, B: video.

An optimal parameter C with respect to the evaluation measure Mean Average Precision (MAP) (see Section 3.1) on the development set was determined for the audio and video modalities. Furthermore, the choice of overlapping vs. non-overlapping sub-windows was evaluated. Results are shown in Figure 5. Generally, overlapping shot sub-windows actually decrease the performance in terms of MAP@20 while this difference is visible, but not as pronounced for MAP@100. As a consequence, 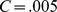 and non-overlapping sub-windows were chosen for the video modality whereas sub-windows with 50% overlap (one second shift) and 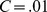 were used for audio. For audio-visual fusion, non-overlapping shot sub-windows were utilized in order to have the same number of predictions per shot.

Regarding the sub-window size, we found that two seconds were actually optimal due to the fact that (i) in most movies, shots are not longer than four seconds on average, limiting the benefit of longer windows, and (ii) shorter windows could not sufficiently increase performance to outweigh the increase in computation time. However, we found that the shot sub-segmentation delivered higher MAP@100 (.451) than simply computing functionals of shots (.437) on the development set.

## Experiments and Results

### 3.1 Evaluation

The primary evaluation measure, as chosen by the MediaEval 2012 Affect Task organizers oriented on the above-named use case [18], is the shot level Mean Average Precision (MAP) at 100. For a single movie, average precision (AP) at 100 is the area under the ‘curve’ that results from considering precision and recall for retrieving the top scored 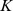 segments, with 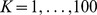. Then, the mean of the APs across all the movies is calculated. Thus, MAP represents the trade-off between recall and precision in a single measure. In addition to MAP@100 we also consider MAP@20 which reflects a use case where the user browses through less shots in the list.

The ground truth label for each of the shot or shot sub-segments is inferred from the violent segment ground truth annotation as follows, in accordance with the MediaEval 2012 campaign. If a shot or shot sub-segment overlaps with a violent segment in some way, the whole shot or whole shot sub-segment is labelled as violent; it is labelled as non-violent otherwise. We would like to note here that a single shot can contain violent and non-violent sub-segments because the boundaries of the violent segments are not aligned to the shot boundaries. Furthermore, a shot can be labeled as violent even if only a small proportion actually contains violence, and vice versa.

In accordance with this ground truth creation procedure, to obtain shot level predictions from shot sub-segment predictions, the scores of the sub-segments that overlap with the shot are averaged. Fusion of audio and video scores is also done by one to one linear averaging of the corresponding shot or shot sub-segment scores from the audio and the video predictions.

Evaluations on the development set are carried out in a 3-fold cross validation. There is no movie overlap between folds and the folds are approximately balanced with respect to violent and non-violent movies, and by year of the movie, in order to ensure that a somewhat representative set is chosen for training in each fold. For the precise fold split of the development set please see Table 1.

### 3.2 System Performance

Firstly different kinds of segmentation, as well as the SMO complexity constant, were validated on the development set by means of a three-fold cross-validation as described above.

Table 4 shows the results obtained with the optimal configuration, on the development and test sets, for audio features, video features, and late audio-visual fusion. Average precisions at 20 and 100 are shown for each movie and MAP is calculated for the development and test set, the latter corresponding to the official score in the MediaEval Affect Task. Foremost, we observe that the average precision strongly varies from movie to movie. In fact, AP@100 is significantly correlated with the violence proportion of the movies (Spearman's 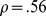 for audio-visual fusion; 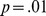). Overall best performance is obtained on *The Bourne Identity* where audio-visual analysis delivers a remarkable AP@20 of .947 (AP@100 = .800), and here modalities seem to be particularly complementary (audio: AP@100 = .639, video: .402). This behaviour can also be found for the ‘next best’ movie *Reservoir Dogs* (audio-visual analysis: MAP@100 = .766). The gain by audio-visual fusion is highest on *Harry Potter V* where neither of the audio nor video modalities can deliver satisfactory performance on their own yet their fusion achieves a MAP@100 of .416. Interestingly, the visual analysis completely fails to retrieve the violent shots from *The Sixth Sense* (MAP@20 = 0, i. e., all of the 20 top-ranked shots are non-violent) while it captures 20 violent shots in the top 20 of *The Wizard of Oz* (MAP@20 = 1), which is especially remarkable since this movie has been artificially coloured, yet no such movie was present in the training set. In total, we have shown the potential of the approach, however, when compared to more complex approaches like [23], which are specifically tailored towards violence detection, the presented approach is outperformed. A direct comparison cannot be performed, because both systems have been modified since the evaluations for the MediaEval workshop, and [23] only reports 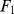-measure instead of MAP.

**Table 4:** Results on MediaEval 2012 Affect Task Data Set: Average precision (AP) and mean average precision (MAP) for violence detection at 20 or 100 top-ranked shots.

	Audio		Video		Audio+Video	
Movie	AP@20	AP@100	AP@20	AP@100	AP@20	AP@100
*Development Set*						
Armageddon	.434	.351	.272	.184	.268	.302
Billy Elliot	.571	.223	.050	.067	.300	.158
Eragon	.553	.532	.502	.372	.642	.412
Harry Potter V	.115	.302	.232	.252	.491	.416
I am Legend	.615	.625	.313	.367	.570	.583
Kill Bill 1	.591	.570	.389	.399	.540	.539
Leon	.480	.434	.145	.174	.543	.439
Midnight Express	.601	.503	.555	.460	.424	.515
Pirates of the Caribbean	.519	.456	.605	.385	.470	.422
Reservoir Dogs	.698	.665	.579	.404	.870	.766
Saving Private Ryan	.550	.530	.507	.416	.600	.553
The Bourne Identity	.753	.639	.636	.402	.947	.800
The Sixth Sense	.361	.187	.000	.028	.083	.096
The Wicker Man	.429	.486	.373	.271	.638	.472
The Wizard of Oz	.369	.274	1.000	.230	.363	.270
Mean	.509	**.452**	**.411**	**.294**	**.517**	**.449**
*Test Set*						
Dead Poets Society	.124	.150	.067	.141	.359	.301
Fight Club	.514	.322	.097	.232	.242	.247
Independence Day	.615	.609	.604	.423	.609	.646
Mean	.418	.360	.256	.265	.403	**.398**

Classification by audio or video features, and late fusion of both. Mean of AP scores across movies in development/test set.

To summarize, we can see evidence for the complementarity of the audio and visual modalities in several movies, especially those where both modalities deliver satisfactory performance on their own. On average, audio-visual fusion outperforms either modality on the test set, but on the development set it cannot outperform audio alone.

## Discussion

### 4.1 Error Analysis

A closer investigation of the results obtained in the MediaEval campaign [17] revealed that the system is quite prone to false positives. Thus, we carried out an additional analysis of the features with respect to the system's predictions (on two second segments). Firstly, to verify that our paradigm for feature relevance analysis captures the features actually taken into account by the classifier, we calculated Spearman's rank correlation coefficient of (i) the feature-wise (absolute) t-statistics as displayed in Figure 1 (i. e., with respect to actual violence), and (ii) the t-statistics with respect to *predicted* violence. We obtained a coefficient of 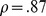 (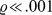), corroborating the validity of our relevance analysis.

Secondly, we investigated the rank correlation of features' t-statistics with respect to false positives vs. true positives, and t-statistics with respect to actual violence; this correlation is considerably lower (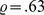), yet significant (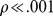), indicating that some features which are descriptive of violence are also prone to leading to false positives. Among these are many of the loudness-related descriptors—e. g., loudness range is among the ‘top 40’ in all three of the lists of relevant features with respect to the label, the prediction, and the false positives. However, other features are not indicative of false positives while being related to the violence label, and being taken into account by the classifier—for instance, the peak distance standard deviation of the spectral centroid contour is ranked # 337 in the list of features relevant for the prediction and has a t-statistic of 14.2 with respect to the violence label, but is at # 3 733 (42-nd last) in the list of features related to false positives. Conversely, some features do not seem to contribute as much to the classifier decision as others, yet are highly indicative of false positives (e. g., the arithmetic mean of first MFCC has a rank of 861 by absolute t-statistic for the violent vs. non-violent label, yet rank 39 for false vs. true positive prediction). Overall, the rank correlation of t-statistics with respect to false positives vs. true positives, and t-statistics with respect to negative vs. positive prediction is ‘only’ 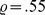.

We repeated this experiment for the video features; the t-statistics of the features with respect to the ground truth and prediction are (rank-)correlated with 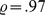, whereas the t-statistics with respect to false positives exhibit a correlation coefficient of 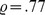 with the t-statistics computed with respect to the ground truth. We conclude that on the one hand, we can build a predictor for false positives that is complementary to the violence predictor itself, and on the other hand, that this opens up promising avenues for wrapper-based feature selection aiming at the reduction of false positives.

### 4.2 Influence of Segmentation

As discussed above, the evaluation according to the official ‘ground truth’ of the MediaEval campaign is oriented on fully automatic segmentation, which does not match the human annotation procedure where annotators segmented the movies into violence and non-violence without using the automatic shot segmentation. Hence, both classifier training and evaluation are ‘noisy’ in the sense that segments containing both violence and non-violence are labeled with only one ‘ground truth’. To provide an upper bound on the performance of our segmental feature extraction, we performed a second sequence of experiments where we do not use the automatic segmentation into shots, but use sub-windows of the segments classified by the human annotators as violent or non-violent. This means that for each training and testing instance a ‘solid ground truth’ exists. For this experiment we also compare the unweighted average recall (UAR) [20] as a measure of overall accuracy in the case of imbalanced class distribution. Results are shown in Table 5. We observe that the segmentation has great influence on the system performance, especially in terms of MAP. There is also a remarkable gain in UAR, but the relative difference is not as strong as for MAP. This indicates that the performance increase is mostly due to the classifier being able to deliver more meaningful scores.

**Table 5:** Influence of segmentation on classifier performance: Shot sub-windows aligned to manual segmentation or to automatic segmentation.

	automatic			manual		
	MAP		UAR	MAP		UAR
	@20	@100		@20	@100	
*Development Set*	.*509*	.*452*	.*682*	.*879*	**.757**	.743
*Test Set*	.*418*	.*360*	.*662*	.*553*	**.598**	.703

Evaluation on MediaEval 2012 Affect Task Data Set, using mean average precision (MAP) or unweighted average recall (UAR).

## Conclusions and Outlook

We have shown an effective, fully automatic approach to violent scenes detection. Evaluating on the official MediaEval campaign data set of original Hollywood movies in full realism, a performance of .398 mean average precision at 100 shots was reached by large-scale brute-forcing of acoustic and visual features, and late fusion. By that, the system achieved competitive results in the official evaluation. In particular, our system does not include any hand-crafting of mid-level classifiers or features, and does not require manual pre-segmentation; yet, including manual pre-segmentation led to a remarkable MAP@100 of up to .598 on the test set.

An in-depth feature analysis has revealed the importance of spectral distribution descriptors as frame-level features, and peak-based functional extraction for the audio channel. From the video channel, very simple descriptors related to color and optical flow have been found relevant. Motivated by the high false positive rate, an error analysis has been carried out and features indicative of false positives have been found which do not overlap with the features which are most important for the classifier's decision; thus, a second predictor could be employed in future work as in [24].

Furthermore, since we found results in terms of mean average precision to vary strongly depending on the parameterization of the feature extraction, we will have to investigate better suited confidence measures from classification than simple hyperplane distances or feature space likelihoods. In particular, cross-database semi-supervised confidence measures as considered by [24] for human affect recognition will be a promising avenue for further leveraging computational intelligence for violent scenes detection. Furthermore, a combination of ‘static’ segmental features with ‘dynamic’ frame-wise classification by (recurrent) neural networks could be used to alleviate the issue of segmentation. Alternatively, unsupervised segmentation techniques could be employed instead of simple fixed length windows.

The benefit of fusion in the presented results is obvious, but very minimal. In future work we need to identify whether more complex fusion techniques or more advanced video descriptors will improve the results, or if simply the visual and acoustic modalities overlap too much, i.e., are too correlated in the given MediaEval 2012 data-set.

From a less technical point of view, in our feature analysis we have found evidence that features correlated to the arousal and valence dimensions are beneficial to violence labeling. Hence, we are confident that in the long run, our findings will deliver another piece of the puzzle that is a generic and holistic statistical model for the affective dimensions of audio-visual recordings. Further unifying the models of human affect recognition, affective sound and video analysis and music mood labeling by joint feature and error analysis in cross-domain setups will be the next step in that direction.
